# Protocol to evaluate sequential electronic health record-based strategies to increase genetic testing for breast and ovarian cancer risk across diverse patient populations in gynecology practices

**DOI:** 10.1186/s13012-023-01308-w

**Published:** 2023-11-06

**Authors:** Heather Symecko, Robert Schnoll, Rinad S. Beidas, Justin E. Bekelman, Daniel Blumenthal, Anna-Marika Bauer, Peter Gabriel, Leland Boisseau, Abigail Doucette, Jacquelyn Powers, Jacqueline Cappadocia, Danielle B. McKenna, Robert Richardville, Lauren Cuff, Ryan Offer, Elizabeth G. Clement, Alison M. Buttenheim, David A. Asch, Katharine A. Rendle, Rachel C. Shelton, Oluwadamilola M. Fayanju, E. Paul Wileyto, Martina Plag, Sue Ware, Lawrence N. Shulman, Katherine L. Nathanson, Susan M. Domchek

**Affiliations:** 1 Perelman School of Medicine, University of Pennsylvania, Philadelphia, PA USA; 2 Basser Center for BRCA, Perelman School of Medicine, University of Pennsylvania, Philadelphia, PA USA; 3 Penn Center for Cancer Care Innovation, Abramson Cancer Center, Penn Medicine, Philadelphia, PA USA; 4 Center for Interdisciplinary Research On Nicotine Addiction, University of Pennsylvania, Philadelphia, PA USA; 5 Feinberg School of Medicine, Northwestern University, Chicago, IL USA; 6 School of Nursing, University of Pennsylvania, Philadelphia, PA USA; 7 Mailman School of Public Health, Columbia University, New York, NY USA; 8 Center for Healthcare Transformation and Innovation, Penn Medicine, Philadelphia, PA USA

**Keywords:** Genetic testing, Behavioral economics, Nudges, Implementation science, Electronic health record, Sequential, Pragmatic, Rapid cycle approaches

## Abstract

**Background:**

Germline genetic testing is recommended by the National Comprehensive Cancer Network (NCCN) for individuals including, but not limited to, those with a personal history of ovarian cancer, young-onset (< 50 years) breast cancer, and a family history of ovarian cancer or male breast cancer. Genetic testing is underused overall, and rates are consistently lower among Black and Hispanic populations. Behavioral economics-informed implementation strategies, or nudges, directed towards patients and clinicians may increase the use of this evidence-based clinical practice.

**Methods:**

Patients meeting eligibility for germline genetic testing for breast and ovarian cancer will be identified using electronic phenotyping algorithms. A pragmatic cohort study will test three sequential strategies to promote genetic testing, two directed at patients and one directed at clinicians, deployed in the electronic health record (EHR) for patients in OB-GYN clinics across a diverse academic medical center. We will use rapid cycle approaches informed by relevant clinician and patient experiences, health equity, and behavioral economics to optimize and de-risk our strategies and methods before trial initiation. Step 1 will send patients messages through the health system patient portal. For non-responders, step 2 will reach out to patients via text message. For non-responders, Step 3 will contact patients’ clinicians using a novel “pend and send” tool in the EHR. The primary implementation outcome is engagement with germline genetic testing for breast and ovarian cancer predisposition, defined as a scheduled genetic counseling appointment. Patient data collected through the EHR (e.g., race/ethnicity, geocoded address) will be examined as moderators of the impact of the strategies.

**Discussion:**

This study will be one of the first to sequentially examine the effects of patient- and clinician-directed strategies informed by behavioral economics on engagement with breast and ovarian cancer genetic testing. The pragmatic and sequential design will facilitate a large and diverse patient sample, allow for the assessment of incremental gains from different implementation strategies, and permit the assessment of moderators of strategy effectiveness. The findings may help determine the impact of low-cost, highly transportable implementation strategies that can be integrated into healthcare systems to improve the use of genomic medicine.

**Trial registration:**

ClinicalTrials.gov. NCT05721326. Registered February 10, 2023. https://www.clinicaltrials.gov/study/NCT05721326

**Supplementary Information:**

The online version contains supplementary material available at 10.1186/s13012-023-01308-w.

Contributions to the literature

•This study will evaluate novel sequential steps to promote the use of genetic testing to determine breast and ovarian cancer predisposition.
•This study will be one of the first to use implementation strategies informed by behavioral economics directed to both patients and clinicians to increase the use of genetic testing for breast and ovarian cancer predisposition across a large health system.
•This study may provide support for low-cost, simple, and scalable approaches to increasing the engagement of at-risk patients with breast and ovarian cancer genetic testing.

## Background

Breast cancer is the most common cancer for women across the world [1–3]. Approximately 300,000 new breast cancer cases were estimated in the USA for 2022, accounting for roughly one-third of new cancer diagnoses among U.S. women [4, 5]. Although less common, ovarian cancer ranks as the deadliest gynecologic cancer, accounting for about 20,000 cases and 13,000 deaths per year [4, 6, 7]. Moreover, substantial health inequities exist for both cancers. Early-stage diagnosis is critical for increasing survival rates [8, 9], but Black women tend to be diagnosed at later stages than white women [7, 10], and mortality rates for both diseases are > 30% higher for Black women as compared to white women [7, 11, 12].

In the past decade, multigene panel tests have been increasingly used to analyze several genes associated with breast and ovarian cancer [13–15]. Genetic testing can inform risk assessment, suggest interventions for risk reduction, and change options for therapy [16]. Multiple FDA approvals for PARP inhibitors for the treatment for BRCA-associated cancer, including early-stage, high-risk breast cancer [17, 18], have changed the landscape with regard to medical intervention [19]. As such, patients [20, 21] and clinicians [20, 22, 23] are generally interested in genetic testing and the National Comprehensive Cancer Network (NCCN) recommends testing for specific groups (e.g., people with personal histories of ovarian cancer) [14]. Unfortunately, there is a clear practice gap in genetic testing uptake, with only about 35% of ovarian cancer patients undergoing testing [24–27]. In assessing two large gynecology practices in our health system, we found higher rates than average of genetic testing among patients diagnosed within the last two years with early-onset breast cancer or triple-negative breast cancer. However, we found very low rates of genetic testing in individuals who were (1) diagnosed with ovarian cancer previously, or (2) had a family history of ovarian cancer or male breast cancer. Testing rates of those with a family history (rather than a personal history) of cancer were less than 15%. Moreover, substantial health inequities exist in testing rates. Eligible Black women are much less likely to be tested than their non-Hispanic white counterparts [11, 25–28], a finding replicated in the analysis of our own data, even with similar referral rates for all patients meeting our eligibility criteria.

Barriers to genomic medicine uptake exist at the system, clinician, and patient levels. The number of available genetic tests is growing exponentially [29, 30], so it can be challenging to integrate genomic data into the electronic health record (EHR) to track genetic test results and facilitate clinical workflows [31–33]. Our health system has tackled this issue by using Health Level 7 (HL7) standardization with labs, genomic indicators, and a precision medicine tab within the EHR [34]. However, due to expanding and changing indications and unclear eligibility criteria [35], clinicians may face challenges in identifying those eligible for genetic testing. Also, they report barriers related to a lack of awareness or training, cost concerns, and busy schedules [33, 35–39], resulting in uncertainty and lower priority for referring patients to genetic counseling. Integrating genomic medicine into the EHR can mitigate some concerns by identifying appropriate patients and guiding clinicians via streamlined workflows [34, 35, 40, 41]. Still, when faced with uncertainty, people often rely on cognitive heuristics to make decisions [42], such as status quo bias, or preferring to maintain the current state over taking action to change [40]. When perceiving unclear eligibility criteria or facing a busy schedule, clinicians may opt to keep things the same to maintain simplicity. The status quo bias can be leveraged by shifting the status quo to default genetics consult orders and emphasizing the availability of EHR-based support systems and clear recommendations for genetic testing. Similarly, default orders can make the referral process easier.

Given the shared decision-making dynamics underlying the choice to pursue genetic testing, patient barriers also must be addressed, including awareness [43–45], access [46], cost concerns [21, 43–47], anxiety about the potential misuse of test results [45, 48, 49], and insurance discrimination [21, 50]. Patients’ decisions are also affected by cognitive heuristics. Omission bias, or focusing on the potential harm of action more than that of inaction [51], plays a key role. Concerns about the implications of test results for patients and their families (such as the need for family members to be tested or fear about how medical professionals or insurance companies may use test results) can trigger omission bias and lead patients to think that getting genetic testing is worse than not pursuing it [52, 53]. These concerns tend to be heightened among members of racial or ethnic minority groups who have been mistreated by the medical system [45, 48, 49]. Although clinician recommendations to pursue genetic testing are one of the strongest predictors of patient willingness to undergo testing [28, 54, 55], clinicians are significantly less likely to recommend it to Black and Hispanic women [28, 54, 56]. Increasing the use of genetic testing requires addressing barriers at multiple levels [57], many of which are salient for patients from minoritized groups. Thus, offering patients the opportunity to be involved in decision-making about genetic testing is crucial for facilitating sustainable and equitable uptake [43].

Leveraging behavioral economic theory to mitigate cognitive heuristics has been effective in promoting evidence-based care and improving patient outcomes [58–62]. Nudges are strategies that make it easier for clinicians and patients to make evidence-based decisions. These can include framing language and/or default options, which subtly change the environment to facilitate evidence-based decisions while still preserving people’s freedom of choice [60, 63]. For instance, emphasizing the ease of genetic testing and the harms of not taking the first steps toward an appointment could mitigate omission bias. For clinicians, nudges can incorporate accountable justification, which requires clinicians to substantiate their decisions when declining a new status quo (in the form of a default order) and can promote self-reflection and higher-value care [64, 65].

Health system nudges have the potential to encourage uptake of genetic testing. These strategies can be automated, are scalable, and could mitigate health inequities [40, 62, 66, 67]. Prior research suggests that clinicians recommend genetic testing less often for non-white patients [28, 54, 56], but sequential dissemination of strategies could expand reach. Initial messages sent via the health system patient portal (MyPennMedicine; MPM) could generate initial interest via a “low-touch” message with minimal costs. Outreach via text messaging, which is more widespread [68], will be able to mitigate inequities resulting from differential patient portal access [69] and facilitate comparisons of outreach strategies. To reduce clinician burden, clinicians will only be contacted in the final phase. Literature suggests that clinician-directed default nudges, which sit atop the nudge intervention ladder, may be the most influential in changing behavior [62, 66].

This study was designed to evaluate the effects of three sequentially delivered patient- and clinician-directed implementation strategies informed by behavioral economics on the scheduling of genetic counseling appointments for patients with breast and ovarian cancer risk. We chose a sequential study design to determine the relative effects of the three strategies. Adaptive designs are gaining in use, including in oncology [70], since they can efficiently help identify the relative benefits of adding components of an intervention in relationship to the overall resources needed to sustain them. Behavioral economics and implementation science can be integrated to maximize the impact and equity of strategies seeking to encourage patients to pursue genetic testing [63].

## Methods

### Study design

**Fig. 1:**
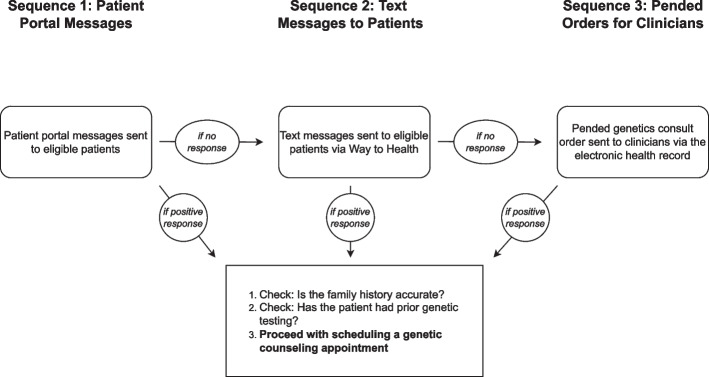
Study design

This pragmatic study will use a non-randomized, sequential adaptive design to examine the effect of patient- and clinician-directed nudges informed by behavioral economics and delivered via the EHR for promoting the use of genetic testing for the identification of breast and ovarian cancer predisposition. Sequences are detailed in Fig. 1. Before the sequences begin, we will use electronic phenotyping procedures to identify a cohort of patients receiving care through two large OB-GYN clinics who are eligible for genetic testing for breast and ovarian cancer predisposition (based on information in the EHR), but who have no documentation of testing in the EHR [71]. Next, in the first sequence, all patients will be contacted via the MyPennMedicine (MPM) patient portal. Messages will be delivered twice, one week apart. The message will include direct information on patients’ cancer risk based on their genetic history, their ability to take action to address their risk, and how to contact the study team to schedule an appointment. Patients can reply directly to the MPM message, call the Cancer Risk Evaluation Program (CREP), or use an outreach-specific email address. Patients who do not engage with the CREP within three weeks will receive the next adaptive sequence, a direct text message targeting omission bias to promote breast and ovarian cancer genetic testing. Again, patients will receive two text messages, a week apart, and will be given three weeks to schedule an appointment. The text message program will be conducted through Penn Way to Health, an evidence-based patient engagement platform [72, 73]. Finally, if patients do not engage with the genetics team, their clinicians will receive an EHR-based nudge targeting status quo bias using the “pend and send” functionality with a pended genetics consult order as a default. A final 6-month observation period will track the scheduling of genetic counseling and genetic testing results for breast and ovarian cancer predisposition following clinician strategy delivery. Patient characteristics (e.g., age, race/ethnicity, nature of genetic risk) will be ascertained from the EHR and explored as moderators of the effectiveness of each sequence to promote genetic testing. This sequential adaptive design will allow for comparisons of which strategies work best across clinics and subgroups of patients.

The primary implementation outcomes will be rates of scheduling and completion of genetic counseling appointments, stratified by factors such as referral clinic and diagnosis, with cumulative nudges representing a time-varying covariate. Decisions to undergo genetic testing in a timely manner are dependent on several factors, including patient preference. Additionally, a counseling discussion can alleviate some patient concerns about testing and may be more acceptable to patients. As such, scheduling the initial genetic counseling appointment was chosen as a primary outcome in addition to appointment completion. Process outcomes will include open rates for the two patient-directed strategies and the proportion of pended orders signed by referring clinicians. We will compare the number of appointments scheduled after each sequence and across patient-level characteristics (e.g., race and ethnicity) and site.

### Study setting, population, and duration

This study will be conducted at gynecologic practices within two Penn Medicine centers: Penn Health for Women Radnor and the Helen O. Dickens Center for Women. These sites serve substantially different patient populations in terms of racial identity and insurance coverage, which may affect testing uptake. Most patients at Radnor are white (83.7% white, 6.5% Black, 3.0% Asian, 6.8% other/unknown), while the Dickens Center predominantly serves Black patients (73.7% Black, 18.9% white, 2.1% Asian, 5.3% other/unknown). Patients seen at these two sites since January 1, 2009 will be selected by an EHR-based algorithm established previously [71] using the following eligibility criteria: (1) serous ovarian cancer diagnosed more than two years prior to study contact; (2) breast cancer diagnosed at under 50 years of age more than two years prior to study contact; (3) triple-negative breast cancer diagnosed at any age more than two years prior to study contact; (4) unaffected individuals reporting a family history of ovarian cancer; (5) unaffected individuals reporting a family history of male breast cancer; and (6) at least two Penn Medicine appointments within the last three years. Utilizing electronic phenotyping in the EHR, participants who have previously received genetic counseling and testing will be excluded. Approximately, 3000 patients at these sites have been identified as eligible for genetic testing for familial high-risk breast and ovarian cancer based on these criteria, and these patients make up the target sample. The clinician sample (*N* = 30) will consist of gynecologists at participating practice sites associated with these patients. It is anticipated that the study will take approximately 18 months to complete. The study was approved by the University of Pennsylvania Institutional Review Board. The trial presents minimal risks to participants, and a waiver of informed consent was approved for all study aims.

### Overview of rapid-cycle approaches and study procedures

The first step to increasing genetic testing is to identify patients who might benefit from it by utilizing updated tools in the EHR. Penn Medicine’s Abramson Cancer Center Electronic Phenotyping Core developed algorithms for identifying patients based on cancer registry data and family history fields in the EHR. These electronic phenotyping procedures were used to identify randomly selected patients to include in pilot testing during the rapid cycle approaches (RCAs).

As a project led by the National Cancer Institute-funded Penn Implementation Science Center in Cancer Control (Penn ISC3; P50CA244690), this study builds on strategies from the center’s prior research [74, 75]. As in prior studies, RCAs to quickly learn and innovate from pilot tests [41, 76–78] were used to de-risk and optimize our nudges as implementation strategies. They also helped us refine our methods based on relevant experiences from clinicians and patient partners to maximize their effect and study efficiency. This work is summarized in Table 1. RCAs involved advisory meetings with experts in behavioral economics, discussions with patient and family partners on the Basser Young Leadership Council, and meetings with clinicians with expertise in breast and ovarian cancer genetic testing. In addition, given the calls to incorporate implementation science and health equity together in genomic medicine [43, 79], experts at integrating these fields provided guidance on study design and message content. We then designed prototype messages and received feedback about content and delivery mechanisms. Finally, two template patient nudges were pilot-tested by randomizing 200 patients to one of two messages that were based on different heuristics. After patients were sent their arm’s respective pilot message via the patient portal and text message, the proportions of patients who engaged with messages, contacted the CREP scheduling staff and scheduled a genetic counseling appointment were assessed. Based on extensive review from partners and pilot test results, the nudges to be implemented in the overall study were designed as follows.

**Table 1:** Rapid cycle approaches to develop, de-risk, and optimize implementation strategies

Domain	Initial approach	Iterative work	Output
Clinician nudge	Best practice alert (BPA) with pended order for genetics counseling Key questions: • What is the best timing and mechanism for sending clinician nudges, since eligible patients won’t be in the clinic very often? • What are the key cognitive heuristics affecting genetic testing ordering?	Method: Meetings with experts in behavioral science, implementation science, health equity, and informatics; discussions with clinicians Key feedback: • Alert fatigue can cause annoyance for care team members • Clinicians preferred a pre-selected recommendation which can be signed efficiently • Status quo bias was a key barrier	“Pend and send” default order for genetic testing with accountable justification for clinicians who decline the order
Patient nudge	Sequential nudges delivered via the patient portal and text message Key questions: • What strategies can be used to overcome inequities in patient portal access? • What are the key cognitive heuristics affecting genetic testing uptake?	Method: Meetings with experts in behavioral science, implementation science and health equity; patient review; pilot tests which contacted 200 patients via the patient portal and text message with two potential nudges Key feedback: • Messages with a clear call to action up front can spur behavior change • Wording changes to increase readability would be valuable • Pilot tests revealed that the message emphasizing ease led to high engagement	Patient portal messages emphasizing ease and text messages emphasizing the importance of taking action to prevent cancer

### Patient nudges (patient portal and text message)

**Fig. 2:**
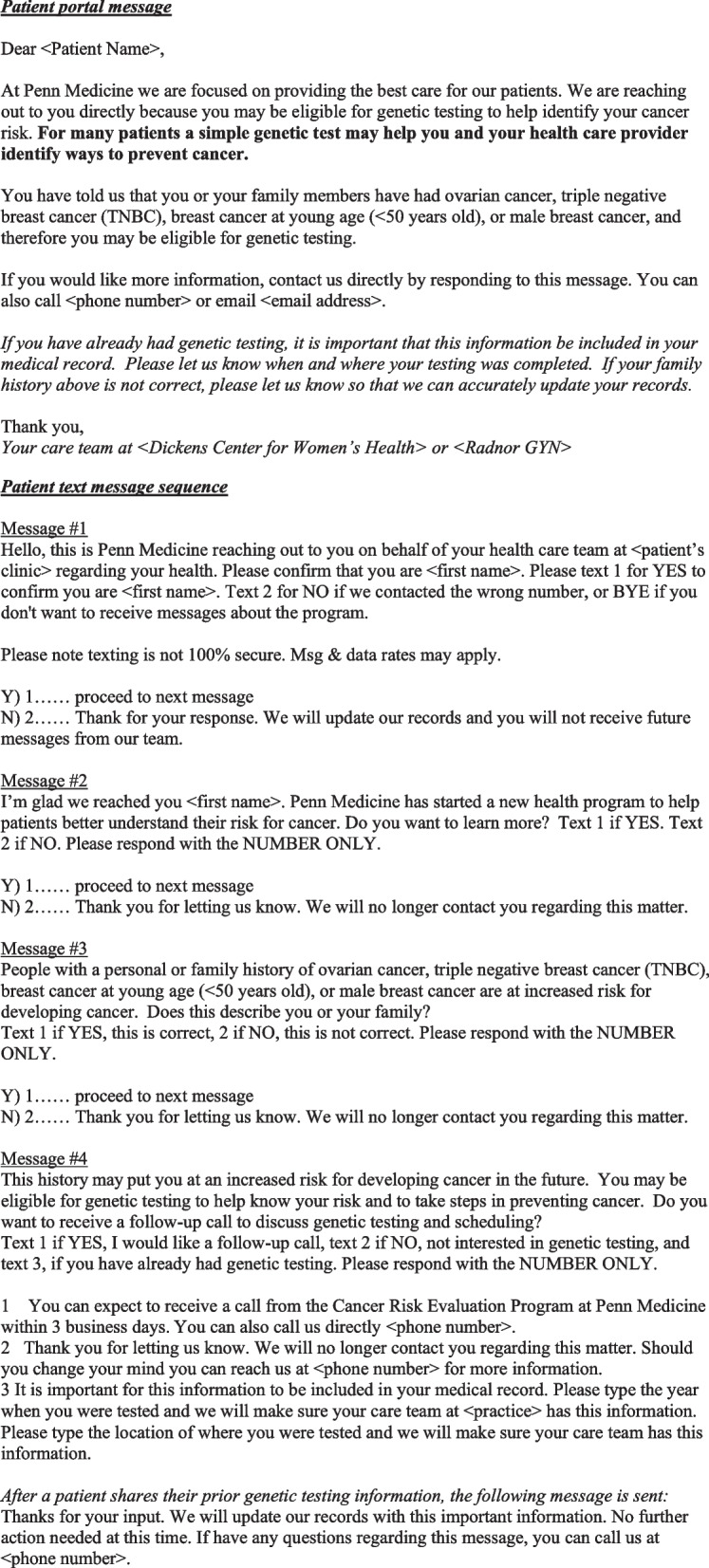
Patient nudges

The patient-directed strategies will be delivered via two mechanisms: the patient portal and text message. Figure 2 shows both patient-directed nudges. The content of the nudges was influenced by past research documenting that patients tend to focus on the potential adverse effects of action vs. inaction [52, 53, 80] and was reviewed and modified by a group of clinicians and patient partners. Eligible patients will initially be contacted via the patient portal. This “low-touch” implementation strategy can provide information about the impact of using patient health system portals to encourage the uptake of genetic testing. If patients do not respond, or if they are identified as not having a patient portal account, they will be moved to the second implementation sequence. In this sequence, patients will be sent text messages using similar content from the first sequence, encouraging them to sign up for genetic counseling appointments. Patients who do not respond to the first two nudges will be identified for the clinician's nudge.

### Clinician nudge

Our preliminary formative work involved the development of prototype messages to integrate as nudges delivered to clinicians through the EHR. As with the patient nudge, we created multiple versions of the nudges and ascertained feedback from partners about the message content and design, as well as the method and timing of delivery. Also, the study team engaged with research from another study promoting genetic testing and adapted the wording and format of a clinician nudge to design a message considered most likely to receive clinician support.

**Fig. 3:**
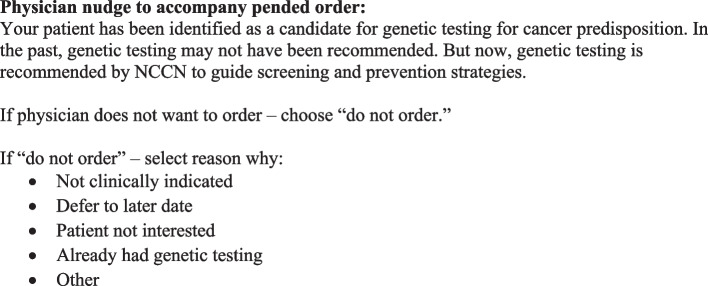
Clinician nudge

Both study sites use Epic (Epic Systems Corporation, Verona, WI) to deliver care. Recent upgrades to Penn’s Epic instance introduced the “pend and send” capability. Using this process, the research team will create a pended order for a genetics consult for eligible patients who did not respond to prior patient-directed strategies. As detailed in Fig. 3, the clinician nudge will include text leveraging status quo bias, a default order for a genetics consult, and a requirement for clinicians to provide accountable justification if they decline the order. Clinicians can efficiently sign orders in the InBasket without needing to open each encounter separately. The consults are then routed directly to the CREP scheduling team, who will contact the patient to schedule an appointment with a genetic counselor. We pilot-tested the “pend and send” mechanism in the RCAs. Clinicians have been engaged at both clinics so that they are aware of this initiative and clinician education has been provided.

### Measures

The primary outcomes are the rates of contacted patients who schedule and complete a genetic counseling appointment within six months after each sequential step, collected via the EHR. As process measures, we will evaluate the proportion of patients who open MPM messages or respond to text messages within one month of receiving them, as well as the proportion of pended orders that clinicians sign within one month to refer patients for a genetics consult. Potential moderating variables will also be collected, including patient demographics (age, race, ethnicity, diagnosis, health insurance, address, and geocoded area as a proxy for neighborhood-level socioeconomic status), clinician data (practice site, years in practice), and practice-level information (community vs. hospital-based setting, urban vs. non-urban location, health insurance mix). These data will be used to describe the sample of participating patients and clinicians and to identify factors that may influence strategy effectiveness. Genetic counseling and testing rates will be evaluated after being stratified by these factors.

### Sample size, power, and statistical analysis

Based on preliminary assessments via electronic phenotyping in the EHR, we have identified a target sample of around 3000 patients (clustered within approximately 30 physicians at the two Penn Medicine sites) who may benefit from genetic testing for familial high-risk breast and ovarian cancer predisposition but have not done so. We calculated power conservatively by assuming correlations of 0 to 0.2, using PASS (Power and Sample Size, NCSS Software, Kaysville, UT). We found our sample gives us 80% power to detect at least a 5% improvement in our cumulative incidence of testing using a two-sided type 1 error rate of 5%, for planned comparisons between each stage in the sequence.

We will analyze the change in the incidence of scheduling counseling appointments across the three sequences (all time to event outcomes) using Cox regression, with variances adjusted for physician clustering. The models will contain time-varying binary predictor terms for each nudge, making adjustments for time in months, and fixed effects for site. We will control for type 1 error inflation by hierarchical testing, starting with the overall model significance, followed by the effect of each strategy. Once we have fitted the main effects model, we will test for each sequence and retain terms if significant (alpha = 5%). Variability in outcomes by sequence and moderators (particularly health equity variables) will be assessed using interaction terms within Cox regression models. We will fit an adjusted Cox regression model using the same approach described in the primary analysis. Covariates of interest available through the EHR will be added to the model, including patient-level (e.g., race), clinician-level (e.g., physician type), and practice-level (e.g., community vs. hospital-based) data.

## Discussion

This study will sequentially test the effects of patient-directed strategies, sent via the patient portal and text message, and EHR-based clinician-directed strategies, sent as “pend and send” default orders, on genetic counseling engagement in gynecology practices at two distinct Penn Medicine clinics. It builds upon Penn ISC3’s prior work [74, 75] implementing nudges to patients and clinicians by extending it to new populations who face additional barriers to engaging in evidence-based clinical practices. Sequential delivery mechanisms can reinforce the additive value of combining outreach via different communication channels, as well as facilitate comparisons between different patient outreach mechanisms. Additionally, the study demonstrates the value of RCAs and pilot testing strategies before implementing them at scale. By addressing barriers and heuristics that affect both patients and clinicians, this multi-level approach may help to define optimal strategies resulting in increased potential for success.

Substantial racial inequities exist in genetic testing, the timeliness of cancer diagnosis, and mortality rates. Implementing these strategies at diverse clinics in this study seeks to mitigate such inequities. While innovative treatments for BRCA-associated cancers (such as PARP inhibitors) are being approved, these innovations can exacerbate racial inequities in downstream outcomes and guideline-concordant receipt of these innovative treatments if genetic testing is not equitably implemented. In combination with existing racial inequities in clinicians’ recommendations for genetic testing, expanding inclusion criteria for genetic testing for all breast cancer patients may widen the divide in genetic testing uptake. Automated outreach via several communication channels aims to alleviate this inequity, and if successful, it can guide future outreach programs to extend health systems’ reach.

Nevertheless, researchers must be mindful of not overloading patients and clinicians with information and support tools. Co-designing strategies with patients and clinicians, switching from a BPA to a “pend and send” default order and the sequential nature of the study has helped mitigate this concern. While the clinics in this study serve diverse patient populations, results may not be generalizable to sites lacking a robust EHR network, capacity for genetic counseling, or leadership support. Finally, this is not a randomized trial. If the implementation strategies demonstrate a positive impact, study results can provide an initial model for encouraging genetic testing uptake and may lead to future large cluster randomized clinical trials focused on scaling these approaches at other Penn Medicine sites and beyond.

### Supplementary Information

**Additional file 1.**

**Additional file 2.** Standards for Reporting Implementation Studies: the StaRI checklist for completion.

## Data Availability

Not applicable.

## Abbreviations

BPA: Best Practice Alert
BRCA: Breast cancer gene
CREP: Cancer Risk Evaluation Program
EHR: Electronic Health Record
FDA: Food and Drug Administration
HL7: Health Level 7
ISC3: Implementation Science Center in Cancer Control
MPM: MyPennMedicine
NCCN: National Comprehensive Cancer Network
NCSS: Number Cruncher Statistical Systems
OB-GYN: Obstetrics and Gynecology
PARP: Poly (ADP-ribose) Polymerase
PASS: Power Analysis & Sample Size
RCA: Rapid cycle approaches
TNBC: Triple negative breast cancer
