# Supplementary material for: Protocol to evaluate sequential electronic health record-based strategies to increase genetic testing for breast and ovarian cancer risk across diverse patient populations in gynecology practices
Source: Implement Sci. 2023 Nov 6;18:57. doi: 10.1186/s13012-023-01308-w (PMC10629034; doi:10.1186/s13012-023-01308-w)
Supplement: Supplementary file 1 — Additional file 1. [file 13012_2023_1308_MOESM1_ESM.pdf]

Institutional Review Board
3600 Civic Center Blvd., 9th Floor
Philadelphia, PA 19104
Phone: 215-573-2540
(Federalwide Assurance # 00004028)

DATE:	31-Aug-2022
TO:	Susan M Domchek
CC:	Symecko, Heather L

RE:	
IRB PROTOCOL#:	851980
PROTOCOL TITLE:	Sequential EHR based interventions to increase genetic testing for breast and ovarian cancer predisposition across diverse patient populations in gynecology practices at Penn Medicine
SPONSOR:	NO SPONSOR NUMBER
REVIEW BOARD:	IRB #8

IRB SUBMISSION: NOTICE OF APPROVAL

Dear Dr. Domchek,

The above referenced protocol was reviewed and approved by the Institutional Review Board using the expedited procedure set forth in 45 CFR 46.110 on 30-Aug-2022. This study has been determined to pose minimal risk to subjects and be eligible for expedited review category(ies) 5, 7.

Consistent with the regulations set forth in 45 CFR 46.109(f), continuing review of this research is not required. IRB approval of this protocol will not expire and continuing review applications should not be submitted. However, you are still required to submit modifications and reportable events to the IRB for review.

The documents included with the application noted below are approved:
-HSERA Application, confirmation code: dgjgcefe, submitted on 08/29/2022

As part of the initial review, a waiver of informed consent and the HIPAA authorization requirement was granted as authorized by 45 CFR 46.116(d) and 45 CFR 164.512 (i), respectively. An expedited review procedure was used for the HIPAA authorization waiver because the research involves no more than minimal risk to the privacy of the individuals who are the subject of the protected health information for which use or disclosure is being sought.

The review of the research has determined the following:

- An adequate plan has been presented to protect the identifiers from improper use and disclosure;
- An adequate plan to destroy the identifiers at the earliest opportunity consistent with conduct of the research exists, unless there is a health or research justification for retaining the identifiers, or such retention is otherwise required by law; and,
- An adequate written assurance has been provided that the protected health information will not be reused or disclosed to any other person or entity, except as required by law, for authorized oversight of the research project, or for other research for which the use or disclosure of protected health information would be permitted under the law.
- That the research cannot practicably be conducted without the waiver to access and use of the protected health information.

ONGOING REQUIREMENTS:

- You must obtain IRB review and approval under 45 CFR 46 if you make any changes to the protocol, consent form, or any other study documents subject to IRB review requirements. Implementation of any changes cannot occur until IRB approval has been given.
- Reportable event, such as serious adverse events, deviations, potential unanticipated problems, and reports of non-compliance must be reported to the IRB in accordance with Penn IRB SOP RR 404.
- When enrolling subjects at a site covered by the University of Pennsylvania's IRB, a copy of the IRB approved informed consent form with the IRB approved from/to stamp must be used unless a waiver of written documentation of consent has been granted.

COMMITTEE APPROVALS: You are responsible for assuring and maintaining other relevant committee approvals. This human subjects research protocol should not commence until all relevant committee approvals have been obtained.

If your study is funded by an external agency, please retain this letter as documentation of the IRB's determination regarding your proposal.

If you have any questions about the information in this letter, please contact the IRB administrative staff. A full listing of staff members and contact information can be found on our website: http://www.irb.upenn.edu

***This letter constitutes official University of Pennsylvania IRB correspondence. ***
